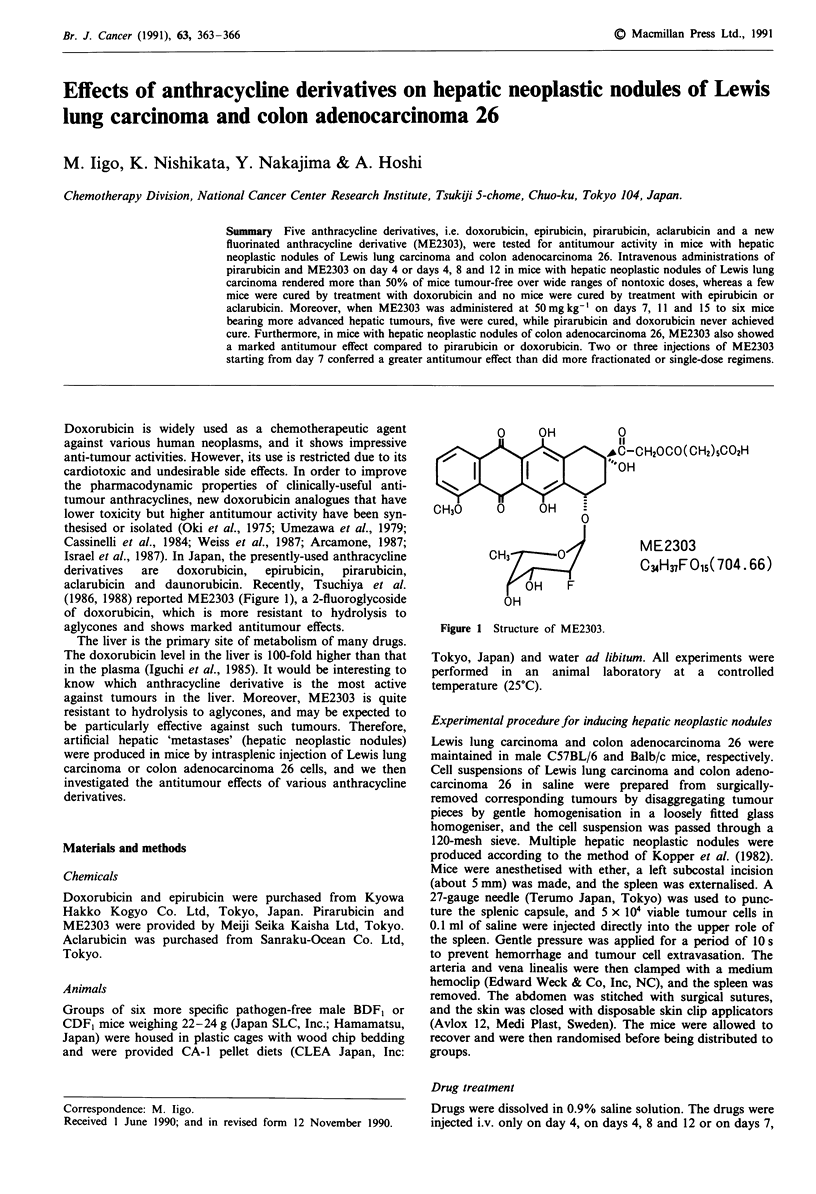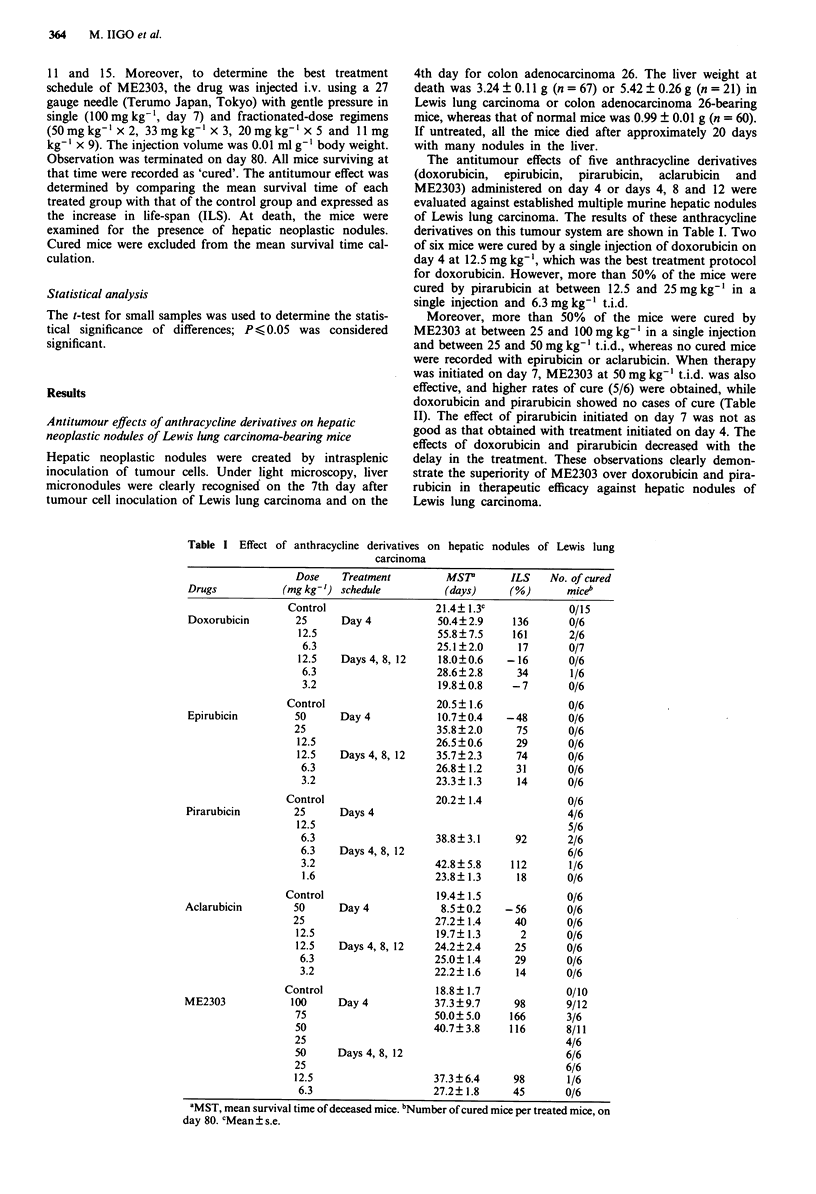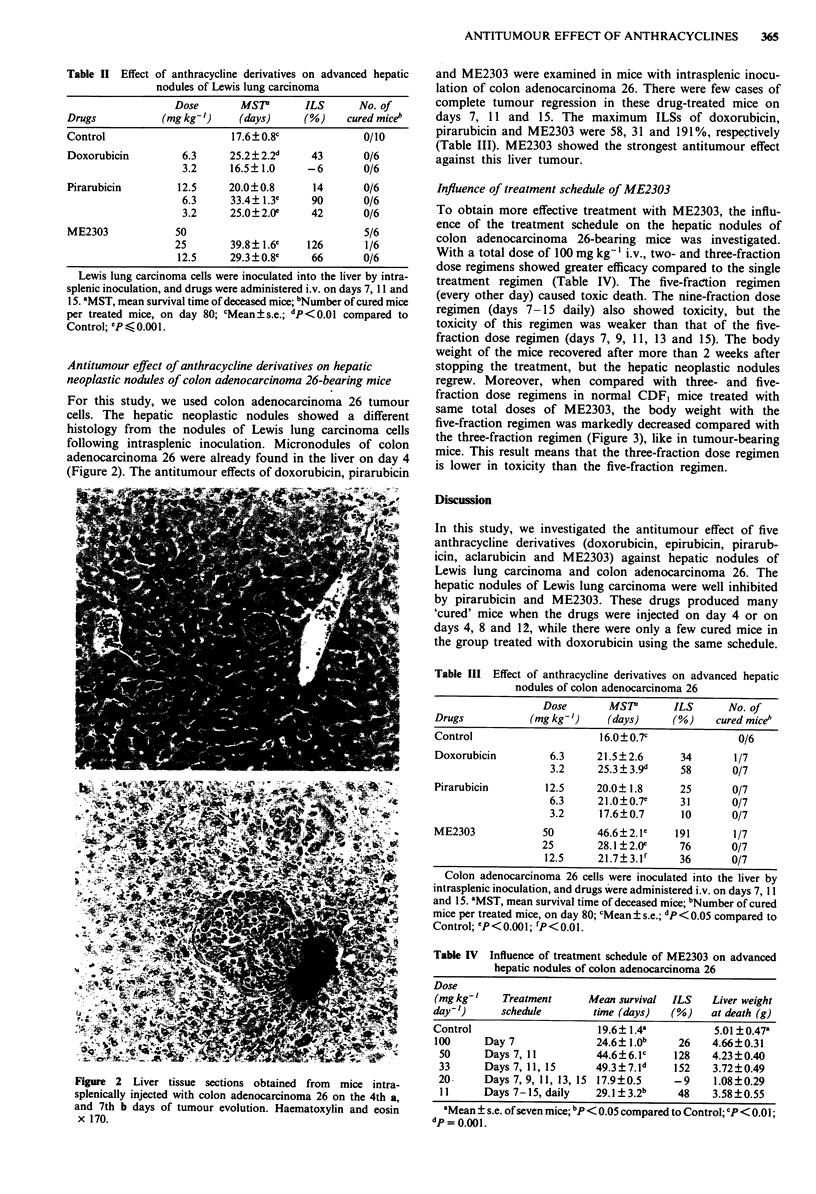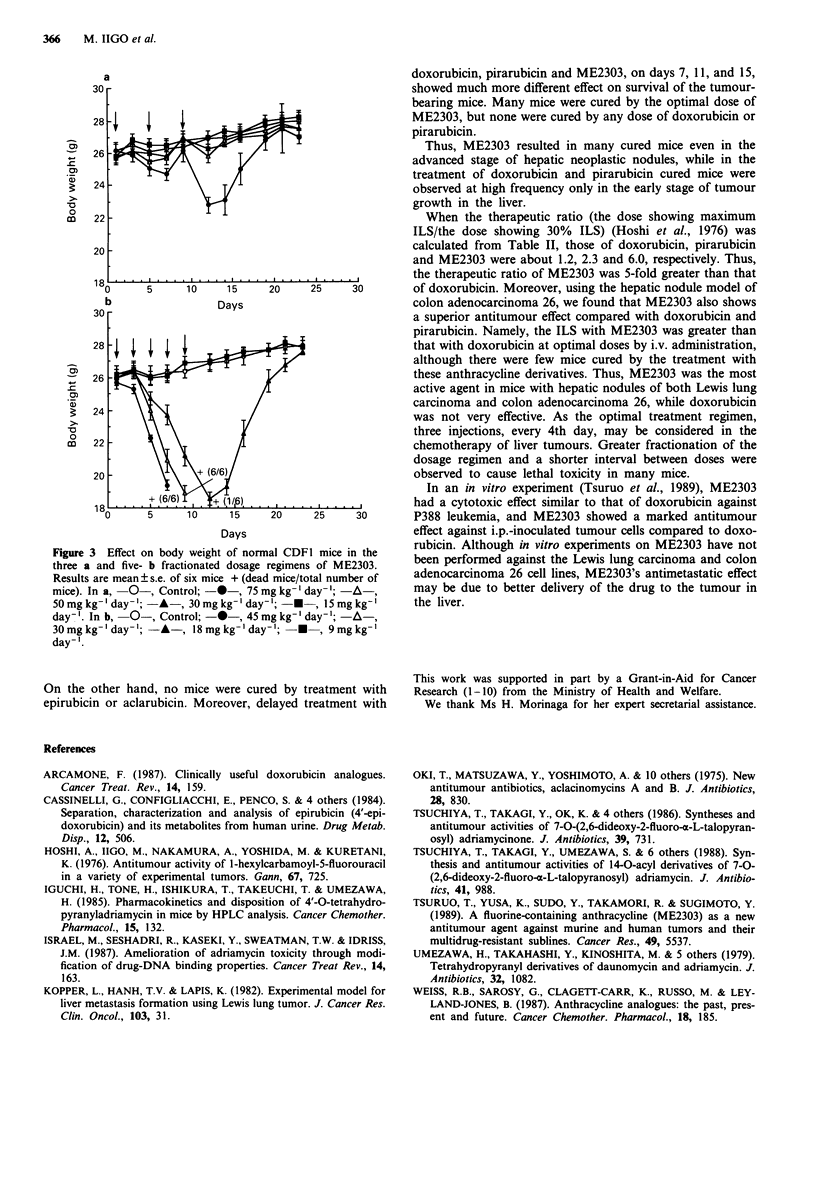# Effects of anthracycline derivatives on hepatic neoplastic nodules of Lewis lung carcinoma and colon adenocarcinoma 26.

**DOI:** 10.1038/bjc.1991.86

**Published:** 1991-03

**Authors:** M. Iigo, K. Nishikata, Y. Nakajima, A. Hoshi

**Affiliations:** Chemotherapy Division, National Cancer Center Research Institute, Tokyo, Japan.

## Abstract

**Images:**


*Br. J. Cancer* (1991), **63**, 363–366

© Macmillan Press Ltd., 1991

### Effects of anthracycline derivatives on hepatic neoplastic nodules of Lewis lung carcinoma and colon adenocarcinoma 26

M. Iigo, K. Nishikata, Y. Nakajima & A. Hoshi

*Chemotherapy Division, National Cancer Center Research Institute, Tsukiji 5-chome, Chuo-ku, Tokyo 104, Japan.*

**Summary** Five anthracycline derivatives, i.e. doxorubicin, epirubicin, pirarubicin, aclarubicin and a new fluorinated anthracycline derivative (ME2303), were tested for antitumour activity in mice with hepatic neoplastic nodules of Lewis lung carcinoma and colon adenocarcinoma 26. Intravenous administrations of pirarubicin and ME2303 on day 4 or days 4, 8 and 12 in mice with hepatic neoplastic nodules of Lewis lung carcinoma rendered more than 50% of mice tumour-free over wide ranges of nontoxic doses, whereas a few mice were cured by treatment with doxorubicin and no mice were cured by treatment with epirubicin or aclarubicin. Moreover, when ME2303 was administered at $50\,mg\,kg^{-1}$ on days 7, 11 and 15 to six mice bearing more advanced hepatic tumours, five were cured, while pirarubicin and doxorubicin never achieved cure. Furthermore, in mice with hepatic neoplastic nodules of colon adenocarcinoma 26, ME2303 also showed a marked antitumour effect compared to pirarubicin or doxorubicin. Two or three injections of ME2303 starting from day 7 conferred a greater antitumour effect than did more fractionated or single-dose regimens.

Doxorubicin is widely used as a chemotherapeutic agent against various human neoplasms, and it shows impressive anti-tumour activities. However, its use is restricted due to its cardiotoxic and undesirable side effects. In order to improve the pharmacodynamic properties of clinically-useful anti-tumour anthracyclines, new doxorubicin analogues that have lower toxicity but higher antitumour activity have been synthesised or isolated (Oki *et al.*, 1975; Umezawa *et al.*, 1979; Cassinelli *et al.*, 1984; Weiss *et al.*, 1987; Arcamone, 1987; Israel *et al.*, 1987). In Japan, the presently-used anthracycline derivatives are doxorubicin, epirubicin, pirarubicin, aclarubicin and daunorubicin. Recently, Tsuchiya *et al.* (1986, 1988) reported ME2303 (Figure 1), a 2-fluoroglycoside of doxorubicin, which is more resistant to hydrolysis to aglycones and shows marked antitumour effects.

The liver is the primary site of metabolism of many drugs. The doxorubicin level in the liver is 100-fold higher than that in the plasma (Iguchi *et al.*, 1985). It would be interesting to know which anthracycline derivative is the most active against tumours in the liver. Moreover, ME2303 is quite resistant to hydrolysis to aglycones, and may be expected to be particularly effective against such tumours. Therefore, artificial hepatic 'metastases' (hepatic neoplastic nodules) were produced in mice by intrasplenic injection of Lewis lung carcinoma or colon adenocarcinoma 26 cells, and we then investigated the antitumour effects of various anthracycline derivatives.

### Materials and methods

### *Chemicals*

Doxorubicin and epirubicin were purchased from Kyowa Hakko Kogyo Co. Ltd, Tokyo, Japan. Pirarubicin and ME2303 were provided by Meiji Seika Kaisha Ltd, Tokyo. Aclarubicin was purchased from Sanraku-Ocean Co. Ltd, Tokyo.

### *Animals*

Groups of six more specific pathogen-free male $BDF_1$ or $CDF_1$ mice weighing 22–24 g (Japan SLC, Inc.; Hamamatsu, Japan) were housed in plastic cages with wood chip bedding and were provided CA-1 pellet diets (CLEA Japan, Inc: Tokyo, Japan) and water *ad libitum*. All experiments were performed in an animal laboratory at a controlled temperature (25°C).

ME2303
$C_{34}H_{37}FO_{15}$(704.66)

**Figure 1** Structure of ME2303.

### *Experimental procedure for inducing hepatic neoplastic nodules*

Lewis lung carcinoma and colon adenocarcinoma 26 were maintained in male C57BL/6 and Balb/c mice, respectively. Cell suspensions of Lewis lung carcinoma and colon adenocarcinoma 26 in saline were prepared from surgically-removed corresponding tumours by disaggregating tumour pieces by gentle homogenisation in a loosely fitted glass homogeniser, and the cell suspension was passed through a 120-mesh sieve. Multiple hepatic neoplastic nodules were produced according to the method of Kopper *et al.* (1982). Mice were anesthetised with ether, a left subcostal incision (about 5 mm) was made, and the spleen was externalised. A 27-gauge needle (Terumo Japan, Tokyo) was used to puncture the splenic capsule, and $5 \times 10^4$ viable tumour cells in 0.1 ml of saline were injected directly into the upper role of the spleen. Gentle pressure was applied for a period of 10 s to prevent hemorrhage and tumour cell extravasation. The arteria and vena linealis were then clamped with a medium hemoclip (Edward Weck & Co, Inc, NC), and the spleen was removed. The abdomen was stitched with surgical sutures, and the skin was closed with disposable skin clip applicators (Avlox 12, Medi Plast, Sweden). The mice were allowed to recover and were then randomised before being distributed to groups.

### *Drug treatment*

Drugs were dissolved in 0.9% saline solution. The drugs were injected i.v. only on day 4, on days 4, 8 and 12 or on days 7,

Correspondence: M. Iigo.
Received 1 June 1990; and in revised form 12 November 1990.

11 and 15. Moreover, to determine the best treatment schedule of ME2303, the drug was injected i.v. using a 27 gauge needle (Terumo Japan, Tokyo) with gentle pressure in single (100 mg kg$^{-1}$, day 7) and fractionated-dose regimens (50 mg kg$^{-1}$ × 2, 33 mg kg$^{-1}$ × 3, 20 mg kg$^{-1}$ × 5 and 11 mg kg$^{-1}$ × 9). The injection volume was 0.01 ml g$^{-1}$ body weight. Observation was terminated on day 80. All mice surviving at that time were recorded as 'cured'. The antitumour effect was determined by comparing the mean survival time of each treated group with that of the control group and expressed as the increase in life-span (ILS). At death, the mice were examined for the presence of hepatic neoplastic nodules. Cured mice were excluded from the mean survival time calculation.

*Statistical analysis*

The *t*-test for small samples was used to determine the statistical significance of differences; $P \leqslant 0.05$ was considered significant.

### Results

*Antitumour effects of anthracycline derivatives on hepatic neoplastic nodules of Lewis lung carcinoma-bearing mice*

Hepatic neoplastic nodules were created by intrasplenic inoculation of tumour cells. Under light microscopy, liver micronodules were clearly recognised on the 7th day after tumour cell inoculation of Lewis lung carcinoma and on the 4th day for colon adenocarcinoma 26. The liver weight at death was 3.24 ± 0.11 g ($n$ = 67) or 5.42 ± 0.26 g ($n$ = 21) in Lewis lung carcinoma or colon adenocarcinoma 26-bearing mice, whereas that of normal mice was 0.99 ± 0.01 g ($n$ = 60). If untreated, all the mice died after approximately 20 days with many nodules in the liver.

The antitumour effects of five anthracycline derivatives (doxorubicin, epirubicin, pirarubicin, aclarubicin and ME2303) administered on day 4 or days 4, 8 and 12 were evaluated against established multiple murine hepatic nodules of Lewis lung carcinoma. The results of these anthracycline derivatives on this tumour system are shown in Table I. Two of six mice were cured by a single injection of doxorubicin on day 4 at 12.5 mg kg$^{-1}$, which was the best treatment protocol for doxorubicin. However, more than 50% of the mice were cured by pirarubicin at between 12.5 and 25 mg kg$^{-1}$ in a single injection and 6.3 mg kg$^{-1}$ t.i.d.

Moreover, more than 50% of the mice were cured by ME2303 at between 25 and 100 mg kg$^{-1}$ in a single injection and between 25 and 50 mg kg$^{-1}$ t.i.d., whereas no cured mice were recorded with epirubicin or aclarubicin. When therapy was initiated on day 7, ME2303 at 50 mg kg$^{-1}$ t.i.d. was also effective, and higher rates of cure (5/6) were obtained, while doxorubicin and pirarubicin showed no cases of cure (Table II). The effect of pirarubicin initiated on day 7 was not as good as that obtained with treatment initiated on day 4. The effects of doxorubicin and pirarubicin decreased with the delay in the treatment. These observations clearly demonstrate the superiority of ME2303 over doxorubicin and pirarubicin in therapeutic efficacy against hepatic nodules of Lewis lung carcinoma.

**Table I** Effect of anthracycline derivatives on hepatic nodules of Lewis lung carcinoma

| *Drugs* | *Dose (mg kg$^{-1}$)* | *Treatment schedule* | *MST*[a] *(days)* | *ILS (%)* | *No. of cured mice*[b] |
|---|---|---|---|---|---|
| | Control | | 21.4±1.3[c] | | 0/15 |
| Doxorubicin | 25 | Day 4 | 50.4±2.9 | 136 | 0/6 |
| | 12.5 | | 55.8±7.5 | 161 | 2/6 |
| | 6.3 | | 25.1±2.0 | 17 | 0/7 |
| | 12.5 | Days 4, 8, 12 | 18.0±0.6 | −16 | 0/6 |
| | 6.3 | | 28.6±2.8 | 34 | 1/6 |
| | 3.2 | | 19.8±0.8 | −7 | 0/6 |
| | Control | | 20.5±1.6 | | 0/6 |
| Epirubicin | 50 | Day 4 | 10.7±0.4 | −48 | 0/6 |
| | 25 | | 35.8±2.0 | 75 | 0/6 |
| | 12.5 | | 26.5±0.6 | 29 | 0/6 |
| | 12.5 | Days 4, 8, 12 | 35.7±2.3 | 74 | 0/6 |
| | 6.3 | | 26.8±1.2 | 31 | 0/6 |
| | 3.2 | | 23.3±1.3 | 14 | 0/6 |
| | Control | | 20.2±1.4 | | 0/6 |
| Pirarubicin | 25 | Days 4 | | | 4/6 |
| | 12.5 | | | | 5/6 |
| | 6.3 | | 38.8±3.1 | 92 | 2/6 |
| | 6.3 | Days 4, 8, 12 | | | 6/6 |
| | 3.2 | | 42.8±5.8 | 112 | 1/6 |
| | 1.6 | | 23.8±1.3 | 18 | 0/6 |
| | Control | | 19.4±1.5 | | 0/6 |
| Aclarubicin | 50 | Day 4 | 8.5±0.2 | −56 | 0/6 |
| | 25 | | 27.2±1.4 | 40 | 0/6 |
| | 12.5 | | 19.7±1.3 | 2 | 0/6 |
| | 12.5 | Days 4, 8, 12 | 24.2±2.4 | 25 | 0/6 |
| | 6.3 | | 25.0±1.4 | 29 | 0/6 |
| | 3.2 | | 22.2±1.6 | 14 | 0/6 |
| | Control | | 18.8±1.7 | | 0/10 |
| ME2303 | 100 | Day 4 | 37.3±9.7 | 98 | 9/12 |
| | 75 | | 50.0±5.0 | 166 | 3/6 |
| | 50 | | 40.7±3.8 | 116 | 8/11 |
| | 25 | | | | 4/6 |
| | 50 | Days 4, 8, 12 | | | 6/6 |
| | 25 | | | | 6/6 |
| | 12.5 | | 37.3±6.4 | 98 | 1/6 |
| | 6.3 | | 27.2±1.8 | 45 | 0/6 |

[a]MST, mean survival time of deceased mice. [b]Number of cured mice per treated mice, on day 80. [c]Mean±s.e.

**Table II** Effect of anthracycline derivatives on advanced hepatic nodules of Lewis lung carcinoma

| *Drugs* | *Dose (mg kg⁻¹)* | *MST*[a] *(days)* | *ILS (%)* | *No. of cured mice*[b] |
|---|---|---|---|---|
| Control | | 17.6±0.8[c] | | 0/10 |
| Doxorubicin | 6.3 | 25.2±2.2[d] | 43 | 0/6 |
| | 3.2 | 16.5±1.0 | −6 | 0/6 |
| Pirarubicin | 12.5 | 20.0±0.8 | 14 | 0/6 |
| | 6.3 | 33.4±1.3[e] | 90 | 0/6 |
| | 3.2 | 25.0±2.0[e] | 42 | 0/6 |
| ME2303 | 50 | | | 5/6 |
| | 25 | 39.8±1.6[e] | 126 | 1/6 |
| | 12.5 | 29.3±0.8[e] | 66 | 0/6 |

Lewis lung carcinoma cells were inoculated into the liver by intrasplenic inoculation, and drugs were administered i.v. on days 7, 11 and 15. [a]MST, mean survival time of deceased mice; [b]Number of cured mice per treated mice, on day 80; [c]Mean±s.e.; [d]$P<0.01$ compared to Control; [e]$P\leqslant0.001$.

### *Antitumour effect of anthracycline derivatives on hepatic neoplastic nodules of colon adenocarcinoma 26-bearing mice*

For this study, we used colon adenocarcinoma 26 tumour cells. The hepatic neoplastic nodules showed a different histology from the nodules of Lewis lung carcinoma cells following intrasplenic inoculation. Micronodules of colon adenocarcinoma 26 were already found in the liver on day 4 (Figure 2). The antitumour effects of doxorubicin, pirarubicin and ME2303 were examined in mice with intrasplenic inoculation of colon adenocarcinoma 26. There were few cases of complete tumour regression in these drug-treated mice on days 7, 11 and 15. The maximum ILSs of doxorubicin, pirarubicin and ME2303 were 58, 31 and 191%, respectively (Table III). ME2303 showed the strongest antitumour effect against this liver tumour.

**Figure 2** Liver tissue sections obtained from mice intrasplenically injected with colon adenocarcinoma 26 on the 4th **a**, and 7th **b** days of tumour evolution. Haematoxylin and eosin × 170.

### *Influence of treatment schedule of ME2303*

To obtain more effective treatment with ME2303, the influence of the treatment schedule on the hepatic nodules of colon adenocarcinoma 26-bearing mice was investigated. With a total dose of 100 mg kg⁻¹ i.v., two- and three-fraction dose regimens showed greater efficacy compared to the single treatment regimen (Table IV). The five-fraction regimen (every other day) caused toxic death. The nine-fraction dose regimen (days 7–15 daily) also showed toxicity, but the toxicity of this regimen was weaker than that of the five-fraction dose regimen (days 7, 9, 11, 13 and 15). The body weight of the mice recovered after more than 2 weeks after stopping the treatment, but the hepatic neoplastic nodules regrew. Moreover, when compared with three- and five-fraction dose regimens in normal $CDF_1$ mice treated with same total doses of ME2303, the body weight with the five-fraction regimen was markedly decreased compared with the three-fraction regimen (Figure 3), like in tumour-bearing mice. This result means that the three-fraction dose regimen is lower in toxicity than the five-fraction regimen.

### Discussion

In this study, we investigated the antitumour effect of five anthracycline derivatives (doxorubicin, epirubicin, pirarubicin, aclarubicin and ME2303) against hepatic nodules of Lewis lung carcinoma and colon adenocarcinoma 26. The hepatic nodules of Lewis lung carcinoma were well inhibited by pirarubicin and ME2303. These drugs produced many 'cured' mice when the drugs were injected on day 4 or on days 4, 8 and 12, while there were only a few cured mice in the group treated with doxorubicin using the same schedule.

**Table III** Effect of anthracycline derivatives on advanced hepatic nodules of colon adenocarcinoma 26

| *Drugs* | *Dose (mg kg⁻¹)* | *MST*[a] *(days)* | *ILS (%)* | *No. of cured mice*[b] |
|---|---|---|---|---|
| Control | | 16.0±0.7[c] | | 0/6 |
| Doxorubicin | 6.3 | 21.5±2.6 | 34 | 1/7 |
| | 3.2 | 25.3±3.9[d] | 58 | 0/7 |
| Pirarubicin | 12.5 | 20.0±1.8 | 25 | 0/7 |
| | 6.3 | 21.0±0.7[e] | 31 | 0/7 |
| | 3.2 | 17.6±0.7 | 10 | 0/7 |
| ME2303 | 50 | 46.6±2.1[e] | 191 | 1/7 |
| | 25 | 28.1±2.0[e] | 76 | 0/7 |
| | 12.5 | 21.7±3.1[f] | 36 | 0/7 |

Colon adenocarcinoma 26 cells were inoculated into the liver by intrasplenic inoculation, and drugs were administered i.v. on days 7, 11 and 15. [a]MST, mean survival time of deceased mice; [b]Number of cured mice per treated mice, on day 80; [c]Mean±s.e.; [d]$P<0.05$ compared to Control; [e]$P<0.001$; [f]$P<0.01$.

**Table IV** Influence of treatment schedule of ME2303 on advanced hepatic nodules of colon adenocarcinoma 26

| *Dose (mg kg⁻¹ day⁻¹)* | *Treatment schedule* | *Mean survival time (days)* | *ILS (%)* | *Liver weight at death (g)* |
|---|---|---|---|---|
| Control | | 19.6±1.4[a] | | 5.01±0.47[a] |
| 100 | Day 7 | 24.6±1.0[b] | 26 | 4.66±0.31 |
| 50 | Days 7, 11 | 44.6±6.1[c] | 128 | 4.23±0.40 |
| 33 | Days 7, 11, 15 | 49.3±7.1[d] | 152 | 3.72±0.49 |
| 20 | Days 7, 9, 11, 13, 15 | 17.9±0.5 | −9 | 1.08±0.29 |
| 11 | Days 7–15, daily | 29.1±3.2[b] | 48 | 3.58±0.55 |

[a]Mean±s.e. of seven mice; [b]$P<0.05$ compared to Control; [c]$P<0.01$; [d]$P=0.001$.

**Figure 3** Effect on body weight of normal CDF1 mice in the three **a** and five- **b** fractionated dosage regimens of ME2303. Results are mean ± s.e. of six mice + (dead mice/total number of mice). In **a**, —○—, Control; —●—, 75 mg kg$^{-1}$ day$^{-1}$; —△—, 50 mg kg$^{-1}$ day$^{-1}$; —▲—, 30 mg kg$^{-1}$ day$^{-1}$; —■—, 15 mg kg$^{-1}$ day$^{-1}$. In **b**, —○—, Control; —●—, 45 mg kg$^{-1}$ day$^{-1}$; —△—, 30 mg kg$^{-1}$ day$^{-1}$; —▲—, 18 mg kg$^{-1}$ day$^{-1}$; —■—, 9 mg kg$^{-1}$ day$^{-1}$.

On the other hand, no mice were cured by treatment with epirubicin or aclarubicin. Moreover, delayed treatment with doxorubicin, pirarubicin and ME2303, on days 7, 11, and 15, showed much more different effect on survival of the tumour-bearing mice. Many mice were cured by the optimal dose of ME2303, but none were cured by any dose of doxorubicin or pirarubicin.

Thus, ME2303 resulted in many cured mice even in the advanced stage of hepatic neoplastic nodules, while in the treatment of doxorubicin and pirarubicin cured mice were observed at high frequency only in the early stage of tumour growth in the liver.

When the therapeutic ratio (the dose showing maximum ILS/the dose showing 30% ILS) (Hoshi *et al.*, 1976) was calculated from Table II, those of doxorubicin, pirarubicin and ME2303 were about 1.2, 2.3 and 6.0, respectively. Thus, the therapeutic ratio of ME2303 was 5-fold greater than that of doxorubicin. Moreover, using the hepatic nodule model of colon adenocarcinoma 26, we found that ME2303 also shows a superior antitumour effect compared with doxorubicin and pirarubicin. Namely, the ILS with ME2303 was greater than that with doxorubicin at optimal doses by i.v. administration, although there were few mice cured by the treatment with these anthracycline derivatives. Thus, ME2303 was the most active agent in mice with hepatic nodules of both Lewis lung carcinoma and colon adenocarcinoma 26, while doxorubicin was not very effective. As the optimal treatment regimen, three injections, every 4th day, may be considered in the chemotherapy of liver tumours. Greater fractionation of the dosage regimen and a shorter interval between doses were observed to cause lethal toxicity in many mice.

In an *in vitro* experiment (Tsuruo *et al.*, 1989), ME2303 had a cytotoxic effect similar to that of doxorubicin against P388 leukemia, and ME2303 showed a marked antitumour effect against i.p.-inoculated tumour cells compared to doxorubicin. Although *in vitro* experiments on ME2303 have not been performed against the Lewis lung carcinoma and colon adenocarcinoma 26 cell lines, ME2303's antimetastatic effect may be due to better delivery of the drug to the tumour in the liver.

This work was supported in part by a Grant-in-Aid for Cancer Research (1–10) from the Ministry of Health and Welfare.
We thank Ms H. Morinaga for her expert secretarial assistance.

### References

ARCAMONE, F. (1987). Clinically useful doxorubicin analogues. *Cancer Treat. Rev.*, **14**, 159.

CASSINELLI, G., CONFIGLIACCHI, E., PENCO, S. & 4 others (1984). Separation, characterization and analysis of epirubicin (4′-epi-doxorubicin) and its metabolites from human urine. *Drug Metab. Disp.*, **12**, 506.

HOSHI, A., IIGO, M., NAKAMURA, A., YOSHIDA, M. & KURETANI, K. (1976). Antitumour activity of 1-hexylcarbamoyl-5-fluorouracil in a variety of experimental tumors. *Gann*, **67**, 725.

IGUCHI, H., TONE, H., ISHIKURA, T., TAKEUCHI, T. & UMEZAWA, H. (1985). Pharmacokinetics and disposition of 4′-O-tetrahydro-pyranyladriamycin in mice by HPLC analysis. *Cancer Chemother. Pharmacol.*, **15**, 132.

ISRAEL, M., SESHADRI, R., KASEKI, Y., SWEATMAN, T.W. & IDRISS, J.M. (1987). Amelioration of adriamycin toxicity through modification of drug-DNA binding properties. *Cancer Treat Rev.*, **14**, 163.

KOPPER, L., HANH, T.V. & LAPIS, K. (1982). Experimental model for liver metastasis formation using Lewis lung tumor. *J. Cancer Res. Clin. Oncol.*, **103**, 31.

OKI, T., MATSUZAWA, Y., YOSHIMOTO, A. & 10 others (1975). New antitumour antibiotics, aclacinomycins A and B. *J. Antibiotics*, **28**, 830.

TSUCHIYA, T., TAKAGI, Y., OK, K. & 4 others (1986). Syntheses and antitumour activities of 7-O-(2,6-dideoxy-2-fluoro-α-L-talopyranosyl) adriamycinone. *J. Antibiotics*, **39**, 731.

TSUCHIYA, T., TAKAGI, Y., UMEZAWA, S. & 6 others (1988). Synthesis and antitumour activities of 14-O-acyl derivatives of 7-O-(2,6-dideoxy-2-fluoro-α-L-talopyranosyl) adriamycin. *J. Antibiotics*, **41**, 988.

TSURUO, T., YUSA, K., SUDO, Y., TAKAMORI, R. & SUGIMOTO, Y. (1989). A fluorine-containing anthracycline (ME2303) as a new antitumour agent against murine and human tumors and their multidrug-resistant sublines. *Cancer Res.*, **49**, 5537.

UMEZAWA, H., TAKAHASHI, Y., KINOSHITA, M. & 5 others (1979). Tetrahydropyranyl derivatives of daunomycin and adriamycin. *J. Antibiotics*, **32**, 1082.

WEISS, R.B., SAROSY, G., CLAGETT-CARR, K., RUSSO, M. & LEYLAND-JONES, B. (1987). Anthracycline analogues: the past, present and future. *Cancer Chemother. Pharmacol.*, **18**, 185.